# Novel Fusion of *MYST/Esa1*-Associated Factor 6 and *PHF1* in Endometrial Stromal Sarcoma

**DOI:** 10.1371/journal.pone.0039354

**Published:** 2012-06-22

**Authors:** Ioannis Panagopoulos, Francesca Micci, Jim Thorsen, Ludmila Gorunova, Anne Mette Eibak, Bodil Bjerkehagen, Ben Davidson, Sverre Heim

**Affiliations:** 1 Section for Cancer Cytogenetics, Institute for Medical Informatics, The Norwegian Radium Hospital, Oslo University Hospital, Oslo, Norway; 2 Centre for Cancer Biomedicine, Faculty of Medicine, University of Oslo, Oslo, Norway; 3 Department of Pathology, The Norwegian Radium Hospital, Oslo University Hospital, Oslo, Norway; 4 Faculty of Medicine, University of Oslo, Oslo, Norway; University Medical Centre Utrecht, The Netherlands

## Abstract

Rearrangement of chromosome band 6p21 is recurrent in endometrial stromal sarcoma (ESS) and targets the *PHF1* gene. So far, *PHF1* was found to be the 3′ partner in the *JAZF1-PHF1* and *EPC1-PHF1* chimeras but since the 6p21 rearrangements involve also other chromosomal translocation partners, other *PHF1*-fusions seem likely. Here, we show that *PHF1* is recombined with a novel fusion partner, *MEAF6* from 1p34, in an ESS carrying a t(1;6)(p34;p21) translocation as the sole karyotypic anomaly. 5′-RACE, RT-PCR, and sequencing showed the presence of an *MEAF6-PHF1* chimera in the tumor with exon 5 of *MEAF6* being fused in-frame to exon 2 of *PHF1* so that the entire *PHF1* coding region becomes the 3′ terminal part of the *MEAF6-PHF1* fusion. The predicted fusion protein is composed of 750 amino acids and contains the histone acetyltransferase subunit NuA4 domain of MEAF6 and the tudor, PHD zinc finger, and MTF2 domains of PHF1. Although the specific functions of the MEAF6 and PHF1 proteins and why they are targeted by a neoplasia-specific gene fusion are not directly apparent, it seems that rearrangement of genes involved in acetylation (*EPC1*, *MEAF6*) and methylation (*PHF1*), resulting in aberrant gene expression, is a common theme in ESS pathogenesis.

## Introduction

Endometrial stromal sarcomas (ESS) are rare malignancies that account for less than 10% of uterine sarcomas [1]. Cytogenetic analysis has been reported in 47 cases of ESS and has revealed three recurrent karyotypic features: 1) Chromosomal translocation t(7;17)(p15∼p21;q12∼q21) in 12 cases (25.5%); 2) Rearrangement of the short arm of chromosome 6, particularly band 6p21, in 12 cases (25.5%); and 3) Chromosomal translocation t(10;17)(q22;p13) in 10 cases (21%) [2], [3]. A recent study showed that tumors having the t(10;17)(q22;p13), which gives rise to *YWHAE-FAM22A* or *YWHAE-FAM22B* fusion genes, have gene expression profiles different from those normally seen in ESS [3]. Current terminology refers to this former subset of ESS as high grade sarcoma NOS [4].

Molecular studies of the t(7;17) have shown that the translocation leads to the fusion of two zinc finger genes, *JAZF1* and *SUZ12* (also named *JJAZ1*) [5]. Subsequent studies of larger tumor series using RT-PCR and fluorescence in situ hybridization (FISH) have shown the occurrence of the *JAZF1/SUZ12* fusion gene not only in ESS but also in endometrial stromal nodules and, less frequently, in undifferentiated endometrial sarcomas [6], [7], [8], [9].

The translocations giving rise to 6p21 rearrangements have differed among ESS tumors. Chromosome 7 has been involved the most with breaks mapping to bands 7p22, 7p21, 7q11, 7q21, and 7q34, the chromosome bands 3p13 and 3q29 were reported to be involved in two tumors each, a der(15)t(6;15)(p21;p12) was found in one tumor, and an add(6)(p21) was described in yet another case [2]. Micci et al [10] showed that in ESS with 6p21 aberrations the target gene is *PHF1* which codes for a protein with significant sequence similarities to Drosophila Polycomblike. This gene was reported to become the 3′partner in two chimeras: a *JAZF1-PHF1* found in two tumors showing a 6p;7p-rearrangement, and an *EPC1-PHF1* (*EPC1* is located at 10p11) in a third tumor with a 6;10;10-translocation as the sole karyotypic abnormality [10]. *JAZF1-PHF1* fusion genes have since been described in 5 additional ESS [11], [12] and *EPC1-PHF1* in 3 more ESS, 2 uterine and 1 extrauterine [11]. *PHF1* rearrangements were found in 5 additional ESS but with unknown partner genes [11]. The consistent involvement of *PHF1* suggests that this gene plays an important role in the development of a subset of ESS. However, both the cytogenetic and molecular data suggest the existence of so-far unknown, additional partner genes for *PHF1* rearrangements. In the present study, we describe an ESS in which *PHF1* was recombined with a novel fusion partner, *MEAF6*, from 1p34.

## Results

### G-banding and FISH Findings

**Figure 1:**
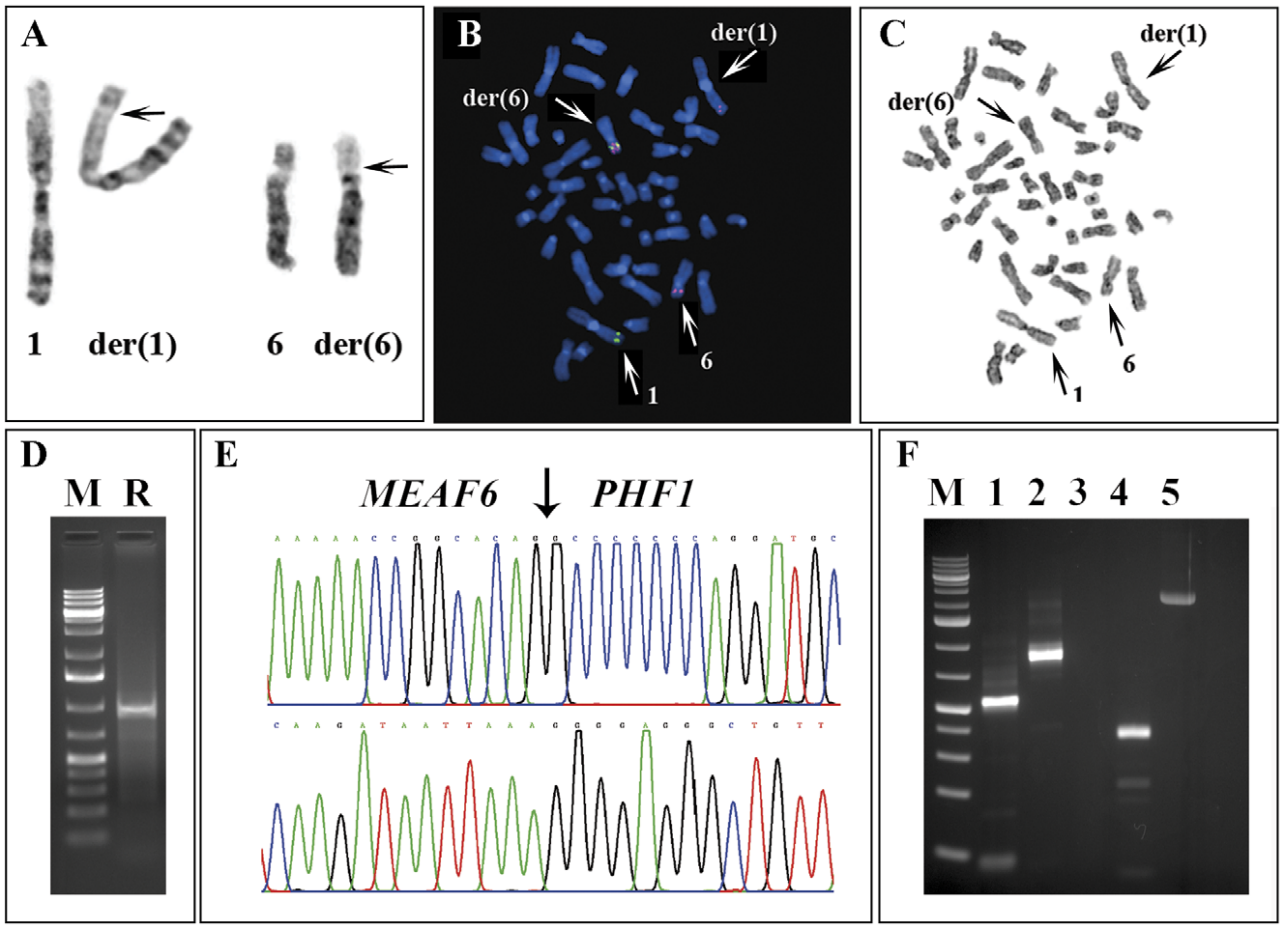
Cytogenetic, FISH, and PCR analyses of the metastasis from the endometrial stromal sarcoma. A) Partial karyotype showing chromosome aberrations der(1)t(1;6)(p34;p21) and der(6)t(1;6)(p34;p21) together with the corresponding normal chromosomes; breakpoint position are indicated by arrows. B) FISH using BAC RP11-508M23 (green signal) from 1p34 containing the *MEAF6* gene and a pool of the RP11-600P03 and RP11-436J22 BACs (red signal) from 6p21 containing the *PHF1* gene. A part of the probe from 6p21 (red signal) has moved to the derivative chromosome 1, while the entire probe containing *MEAF6* has moved to the derivative chromosome 6. The data suggest that the functional fusion gene is generated on the der(6). C) G-banding of the metaphase spread shown in (B). D) Amplification of a 1 kb cDNA in the 5′-RACE analysis (R) using reverse PHF1-721R and PHF1-526R primers and the universal forward primers. E) Partial sequence chromatograms of the 1 kb cDNA fragment showing the junctions (arrow) of *MEAF6-PHF1* chimeric transcript (upper) and genomic hybrid DNA fragment (lower). F) RT-PCR and genomic PCR using specific MEAF6 and PHF1 primers. Lane 1: Amplification of *MEAF6-PHF1* cDNA fragment with MEAF6-322F/PHF1-380R primers, lane 2: Amplification of *MEAF6* transcript with MEAF6-15F/MEAF6-700R, lane 3: PHF1-18F/MEAF6-729R primer set did not amplify the reciprocal PHF1-MEAF6 cDNA, lane 4: Amplification of *PHF1* transcript with PHF1-18F/PHF1-327R primers, lane 5: Amplification of *MEAF6-PHF1* genomic hybrid DNA fragment with MEAF6-371F/PHF1-302R primer combination. M, 1 kb DNA ladder.

The G-banding and FISH analyses yielded the karyotype 46,XX,t(1;6)(p32∼34;p21) (Figure 1A). When metaphase spreads were hybridized with the *PHF1*-specific probe, a split signal was seen, indicating that the translocation breakpoint on chromosome 6 was within the *PHF1* locus (Figure 1B). FISH with a BAC probe containing the *MEAF6* locus on chromosome 1 showed that *MEAF6* had moved to the derivative chromosome 6 (Figure 1B).

### Molecular Genetic Findings

5′-RACE methodology amplified a single 1 kb fragment (Figure 1D). Sequence analysis showed that the fragment was a hybrid cDNA product in which exon 5 of the *MEAF6* gene (nt 550 in NM_022756 version 4), from sub-band 1p34.3, was fused in-frame to exon 2 of *PHF1* (nt 200 in NM_024165 version 2), from sub-band 6p21.32 (Figure 1E). Subsequently, RT-PCR with the MEAF6-322F/PHF1-380R primer combination amplified a 435 bp cDNA fragment, whereas amplification with the MEAF6-460F/PHF1-327R primer combination generated a 241 bp cDNA fragment (Figure 1F). The fragments were analyzed by direct sequencing which verified the presence of a *MEAF6-PHF1* chimeric transcript. A reciprocal *PHF1-MEAF6* cDNA fragment, looked for using two primer sets (PHF1-18F/MEAF-729R and PHF1-136F/MEAF6-700R), was not amplified. Genomic PCR amplified a 2.5 kb genomic fragment (Figure 1F). Partial sequencing showed that the breakpoints were located in intron 5 of *MEAF6* and intron 1 of *PHF1* (Figure 1E). A reciprocal *PHF1/MEAF6* genomic fragment using *PHF1* forward primers in exon 1 and *MEAF6* reverse primers in exon 6 was not amplified (data not shown).

## Discussion

In the present study we showed that *PHF1* was rearranged and fused to a novel partner gene, *MEAF6*, as the result of a t(1;6)(p34;p21) occurring as the sole chromosomal aberration in an ESS. This recombination has extensive similarities with the other two *PHF1* chimeras, *JAZF1-PHF1* and *EPC1-PHF1*, found in ESS [10], [12]. At the genomic level: 1) *MEAF6* and *PHF1* are transcribed in opposite orientations, suggesting that additional genomic events are required for the formation of a functional *MEAF6-PHF1* fusion as has been described also for *JAZF1-PHF1* and *EPC1-PHF1* [10], [12]; 2) the breakpoint in *PHF1* occurred in intron 1 similarly to what happens in the fusions with *JAZF1* and *EPC1*. This intron is 1032 bp long and contains an AluSg repeat; and 3) the reciprocal *PHF1-MEAF6* chimera could not be detected suggesting its absence or that it has undergone complex genetic changes rendering it undetectable by PCR. At the transcriptional level, *MEAF6-PHF1* seemed to be the chimeric transcript of tumorigenic importance since RT-PCR did not amplify the reciprocal *PHF1-MEAF6* transcript. Similarly to what has been described for the *JAZF1-PHF1* and *EPC1-PHF1* fusion genes in ESS, the entire *PHF1* coding region becomes the 3′ terminal part of the *MEAF6-PHF1* fusion which gives a predicted fusion protein of 750 amino acids containing the histone acetyltransferase subunit NuA4 domain of MEAF6 and the tudor, PHD zinc finger and MTF2 domains of PHF1.

The *MEAF6* gene acting as the 5′-partner in the fusion is ubiquitously and abundantly expressed and encodes a protein which is part of the TIP60-ING3, HBO1-ING4/5, and MOZ/MORF histone acetyltransferase (HAT) multi-subunit complexes of the MYST family [13], [14], [15]. The MYST histone acetyltransferases are highly conserved in eukaryotes and carry out a significant proportion of all nuclear acetylation. TIP60-ING3 is responsible for acetylation of histones H4 and H2A, HBO1-ING4/5 for histone H3 and H4 acetylation, and MOZ/MORF histone acetyltransferase for H3 acetylation. They function exclusively in multisubunit protein complexes and play critical roles in gene-specific transcription regulation, DNA damage response and repair, as well as DNA replication [16], [17]. In the TIP60-ING3 complex, MEAF6 physically interacts with the EPC1 protein shown to be fused to the entire coding sequence of *PHF1* in the *EPC1-PHF1* chimera [16], [17].

*PHF1* encodes a Polycomb group (PcG) protein that contains a tudor domain, PHD zinc finger domains, and a polycomb-like MTF2 factor 2 domain [18]. PcG proteins are thought to form a multimeric complex that modifies local chromatin structure and establishes a heritable repression state at particular loci.

Tudor domains of several chromatin related proteins interact with various methylated lysine and arginine residues [19]. The plant homeodomain (PHD) finger is a C4HC3 zinc-finger-like motif found in nuclear proteins thought to be involved in epigenetics and chromatin-mediated transcriptional regulation [20]. Mammalian Polycomb-like protein MTF2/PCL2 forms a complex with Polycomb repressive complex-2 (PRC2) and collaborates with PRC1 to achieve repression of *Hox* gene expression [21].

PHF1 is a component of a histone H3 lysine-27 (H3K27) specific methyltransferase complex and is important for *Hox* gene expression in vivo [22], [23]. Hong et al [24] showed that PHF1 is also recruited to DNA double strand breaks and interacts physically with many proteins which are involved in DNA damage response. Although the specific functions of the MEAF6 and PHF1 in the neoplastic context and why they are involved in an ESS-specific fusion are not directly apparent, one can assume a mechanism similar to the EPC1-PHF1 proposed by Avvakumov and Côté [16], namely that the MEAF6-PHF1 chimeric protein diverts HAT activity towards PHF1’s normal genomic targets. Mistargeted acetylation would lead to loosening up of the heterochromatin, resulting in aberrant gene expression that could eventually lead to a malignant phenotype.

Although the t(1;6)(p34;p21) presented here has never been reported before in ESS, a t(1;6)(p32–33;p21.3) was found as the sole clonal cytogenetic abnormality in what was called a uterine leiomyosarcoma [25]. Additionally, involvement of chromosome arm 1p was reported in four ESS [26], [27], [28], [29]. Since the formation of a functional MEAF6-PHF1 chimera requires complex genomic events, the presence of hidden MEAF-PHF1 fusions in ESS whose karyotypes include 1p rearrangements or other complex and incompletely described abnormalities is possible.

## Materials and Methods

### Case History

A 43-year-old female presented with a tumor in the uterus and a total hysterectomy was performed. The histological diagnosis was endometrial stromal sarcoma and macroscopic evaluation showed multiple nodules in the myometrium and a 13 cm large tumor on the outer aspect of the uterus. After four years of follow-up, metastases in the pelvic region were detected and a resection was made. Seven months after the metastasectomy the patient is in remission.

**Figure 2:**
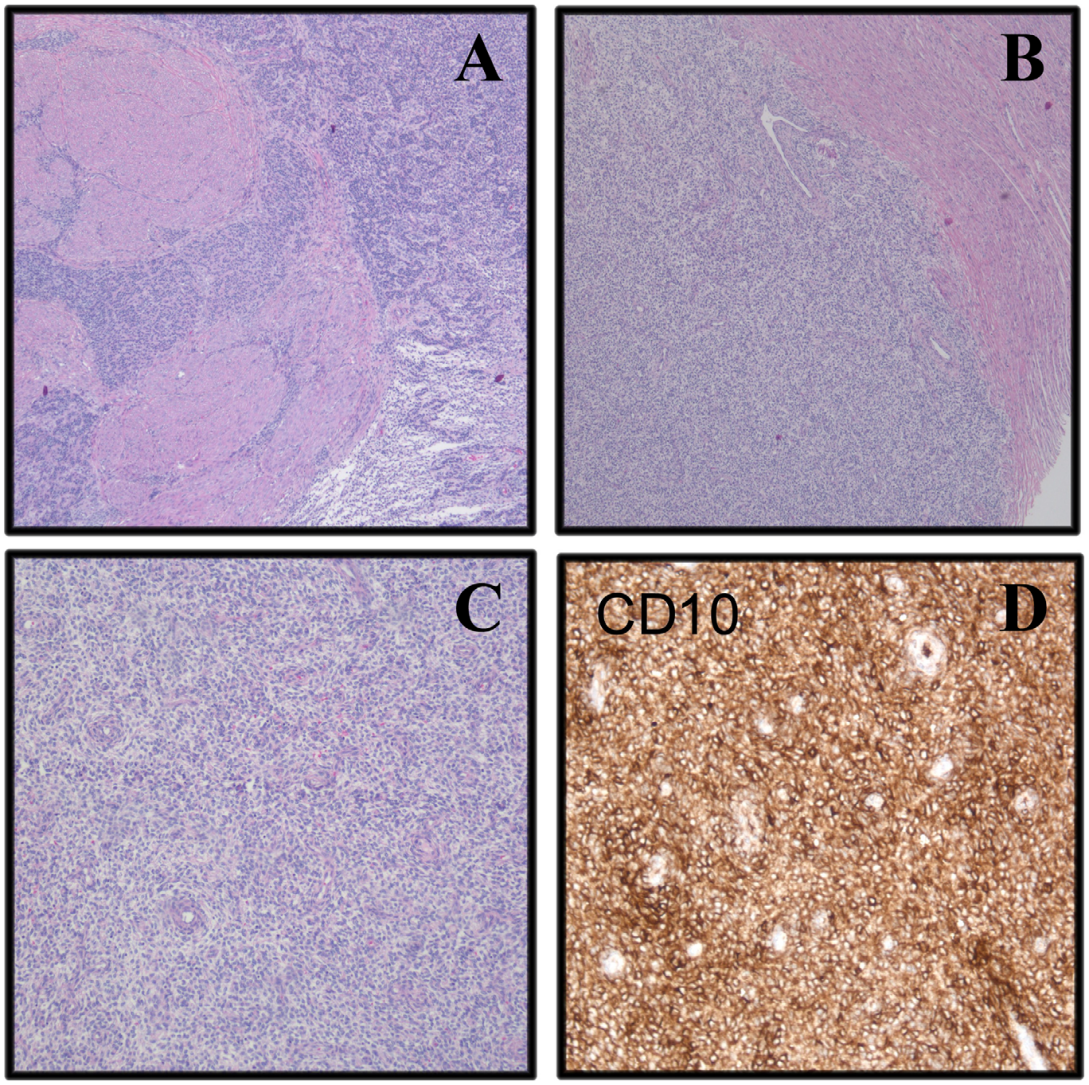
Microscopic examination of the primary endometrial stromal sarcoma. A–C: H&E-stained sections of the tumor (A, B: x50 magnification; C: x100 magnification); D: Positive immunostaining for CD10 (x100 magnification).

Microscopic examination of the primary tumor showed a relatively monotonous, diffusely infiltrating growth pattern with variable cellularity and bland, uniform spindle cells. Small vascular structures were seen. There was no necrosis or vessel infiltration (Figure 2). Immunohistochemical examination showed strong staining for vimentin, CD10, h-caldesmon, PGR and focally for SMA, ER and desmin, but negative results for CD117, S-100, and pan-cytokeratin (AE1/AE3). The metastasis displayed the same morphological appearance.

The study was approved by the Regional komité for medisinsk forskningsetikk Sør- Norge (REK Sør, http://helseforskning.etikkom.no), and written informed consent was obtained from the patient.

### G-banding and Karyotyping

Tumor tissue removed during surgery directed against the metastases was processed for cytogenetic analysis using standard methods [30]. Chromosome preparations made from metaphase cells of a week-old culture were G-banded using Wright stain and karyotyped according to ISCN 2009 guidelines [31].

### Fluorescence in situ Hybridization Analyses

BAC clones were retrieved from the RPCI-11 Human BAC library (BACPAC Resources, http://bacpac.chori.org/home.htm). They were selected according to physical and genetic mapping data on chromosomes 1 and 6 (see below) as reported on the Human Genome Browser at the University of California, Santa Cruz website (May 2004, http://genome.ucsc.edu/). In addition, FISH mapping of the clones on normal controls was performed to confirm their chromosomal location. The clones used were RP11-508M23 mapping to 1p34.3 which contains the *MEAF6* gene, and RP11-600P03 and RP11-436J22 mapping to 6p21.32–6p21.31 for *PHF1*. DNA was extracted and probes were labelled and hybridized as previously described [32]. Chromosome preparations were counterstained with 0.2 µg/ml DAPI and overlaid with a 24×50 mm^2^ coverslip. Fluorescent signals were captured and analyzed using the CytoVision system (Applied Imaging, Newcastle, UK).

### Molecular Genetic Analyses

**Table 1:** Primers used for PCR and sequencing.

Primer	Sequence (5′→3′)
MEAF6-15F	AAC ATG GCG ATG CAC AAC AAG GC
MEAF6-322F	CAT TGG CAG GAG TTC AGG ACC AGC
MEAF6-371F	TGG GAC GGA AAG TGA CAC TTC TCC A
MEAF6-423F	AGC CAG GAG GAC CCT GAG GAT CTG
MEAF6-460F	AGG GAG TGA AAC CTC AGA AGG CTG CT
MEAF6-700R	TGG ATG GCC ACG AAC TCA GGT GA
MEAF6-729R	TCA CAG GCA CTG GGT CTG GGA GAG
PHF1-18F	GCT GCT TTG GCT GCT GCG TCA TA
PHF1-136F	GGG GAC TCG CCT AGG TCT CCT ACG
PHF1-302R	CAT CTT GAC CCT CCC AAA GCC GA
PHF1-327R	AGC CCA TCA GTC CAT CTG GCC AG
PHF1-380R	GGA CCA GAC ACA CCT CCC TAG CAC TG
PHF1-526R	GGC GAC ACT TCT CAC AGC TGA CCA
PHF1-721R	CAG TCC AGC CCC TTC AGT CCA T

#### 5′-Rapid amplification of cDNA ends

The primers used for PCR amplification and sequencing are listed in Table 1. Total RNA and genomic DNA were extracted using Trizol reagent according to the manufacturer’s instructions (Invitrogen). Two µg of total RNA were then used for cDNA preparation and 5′-RACE was performed using the GeneRacer kit (Invitrogen) according to the manufacturer’s protocol. In brief, the ligated RNA was reverse transcribed using Cloned Avian Myeloblastosis Virus reverse transcriptase (Cloned AMV RT) and first round polymerase chain reaction (PCR) was done with the forward GeneRacer 5′Primer and PHF1-721R reverse primer. Second round PCR was performed with the forward GeneRacer 5′Nested Primer and the primer PHF1-526R. For PCR the AccuPrime *Taq* DNA Polymerase High Fidelity was used (Invitrogen). The template (1 µL cDNA) was amplified in a 50 µL volume containing 0.2 µM of each forward and reverse primer, 1×AccuPrime PCR buffer I, and 1.25 U AccuPrime *Taq* DNA Polymerase High Fidelity. One µL of the PCR products was re-amplified in a second PCR using 0.2 µM of each GeneRacer 5′Nested Primer and the reverse primer PHF1-526R. After an initial denaturation for 1 min at 94°C, 30 cycles of 15 s at 94°C, 30 s at 56°C (63°C for nested PCR), and 3 min (1 min for nested PCR) at 68°C were run, followed by a final extension for 5 min at 68°C. Fifteen µL of the PCR products were analyzed by electrophoresis through 1.5% agarose gels, stained with GelRed (Biotium), and photographed. The amplified products were excised from the gel, purified using the Qiagen gel extraction kit (Qiagen), and cloned to pCR4-TOPO vector using TOPO TA Cloning Kits for Sequencing (Invitrogen). Eight colonies were sequenced using the dideoxy procedure with an ABI Prism BigDye terminator v1.1 cycle sequencing kit (PE Applied Biosystems) on the Applied Biosystems Model 3100-Avant DNA sequencing system. The BLAST software (http://www.ncbi.nlm.nih.gov/BLAST/) was used for computer analysis of sequence data.

### PCR Analyses

Two µg of total RNA were reverse-transcribed in a 20 µL reaction volume using iScript Advanced cDNA Synthesis Kit for RT-qPCR according to the manufacturer’s instructions (Biorad). The cDNA was diluted to 100 µL and 2 µL were used as templates in subsequent PCR assays. A one-step PCR was performed for amplification of the *MEAF6/PHF1* and possible reciprocal *PHF1/MEAF6* fusion transcripts as well as normal *MEAF6* and *PHF1* transcripts. The 50 µL PCR volume contained 2 µL of cDNA, 1x LA PCR Buffer II (Mg^2+^ plus), 0.4 mM of each dNTP, 2.5 unit TaKaRa LA *Taq* (TaKaRA), and 0.6 µM of each of the forward and reverse primers. For amplification of the *MEAF6/PHF1* fusion transcript the MEAF6-322F/PHF1-380R and MEAF6-460F/PHF1-327R primer combinations were used. For amplification of the *MEAF6* transcript the primers MEAF-423F/MEAF6-729R were used, and for amplification of PHF1 cDNA the primer set PHF1-18F/PHF1-327R was used. For a possible reciprocal *PHF1/MEAF6* transcript the primer sets PHF1-18F/MEAF-729R and PHF1-136F/MEAF6-700R were used.

For genomic PCR the 50 µL PCR volume had the same composition as above except that 100 ng DNA were used as template. For the detection of genomic MEAF6-PHF1 and PHF1-MEAF6 hybrids the primer sets MEAF6-460F/PHF1-729R and PHF1-136F/−MEAF6-700R were used.

The PCRs were run on a C-1000 Thermal cycler (Biorad). The PCR conditions were: an initial denaturation at 94°C for 1 min, followed by 30 cycles of 7 sec at 99°C and 1 min at 68°C (3 min for genomic PCR), and a final extension for 10 min at 72°C. Two µL of each PCR amplification were run on 1.5% agarose gel, stained with GelRed (Biotium), and photographed.
